# Digital health systems and inequities: exploring climate and environmental impacts

**DOI:** 10.1093/oodh/oqaf034

**Published:** 2025-12-18

**Authors:** Natalie Hammond, Peter Benjamin, Gabrielle Samuel, Javier Elkin, Tanjir Rashid Soron, Richard Holman Matanta, Akaninyene Obot, Carme Carrion

**Affiliations:** Department of Social Care and Social Work, Bonsall Street, Manchester Metropolitan University, Manchester, M15 6GX, UK; Health Enabled, 6 Wherry Road, Cape Town, Western Cape, 7945, South Africa; Department of Global Health and Social Medicine, North East Wing, Bush House, Aldwych, King’s College London, WC2B 4BG London, UK; International Committee of the Red Cross, 19 Avenue de la Paix, 1202 Geneva, Switzerland; Ni Health Ltd & Telepsychiatry Research and Innovation Network Ltd, Level -8, TMC Building, Road No- 52 New Eskaton Road, District/Division Dhaka 1000, Bangladesh; Department of Public Health and Family Medicine, Jl. Perintis Kemerdekaan No. KM. 10, Tamalanrea Indah, Tamalanrea, South Sulawesi, 90245, Indonesia; Department of Agricultural Economics, Nnamdi Azikiwe University, Enugu-Onitsha Expressway, Awka, Anambra State, 420007, Nigeria; eHealth Lab Research Group, eHealth Centre, Universitat Oberta de Catalunya (UOC), Rambla Poblenou 156, 08018 Barcelona, Catalonia, Spain; School of Health Sciences, Universitat de Girona (UdG), Emili Grahit 77, 17071 Girona, Catalonia, Spain

**Keywords:** climate change, environmental harm, digital health, equity, equality

## Abstract

The digital health community has taken an increased interest in climate and environmental issues. At the same time, and even though equity considerations have been central to digital health for decades, equity-focused approaches to climate and the environment have been less considered. In this commentary, we emphasize the need for equity to remain central to digital health as it shifts to address climate and environment-related challenges. We provided four case examples: electronic waste, early warning systems, digital systems infrastructure vulnerability and artificial intelligence resource use, to demonstrate the climate/environment digital health inequity nexus. We stress that, in not attending to this relationship, the digital transition may amplify rather than reduce inequities associated with climate and environmental challenges. We include a call to action for those in the digital health community to include equity considerations in the planning and designing of digital health solutions and systems aimed at addressing such climate and environmental challenges and beyond.

## INTRODUCTION

Healthcare systems around the world are becoming digitalized and the transformative effect of this digitalisation promises to improve universal health coverage, enhance access to healthcare services, provide professionals and communities with better knowledge and access to health information, deliver financial benefits, protect people from health emergencies, raise safety and product performance and improve overall health and wellbeing [1, 2]. At the same time, it is well recognized that digital health solutions must be developed and implemented equitably, meaning there should be an absence of unfair, avoidable or remediable differences among groups of people, whether those groups are defined socially, economically, demographically, or geographically or by other dimensions of inequality (e.g. sex, gender, ethnicity, disability, or sexual orientation) [3]. Nevertheless, unequal access to digital health technologies and/or their necessary infrastructure remains. This amplifies inequity, as this lack of access often affects groups of people who are already systematically socially disadvantaged (e.g. by being poor, female and/or members of a disenfranchised racial, ethnic, or religious group), putting them at further disadvantage with respect to their health [4]. Alongside these inequities, climate change and environmental degradation have already amplified existing equity concerns associated with healthcare delivery, because both climate change and environmental degradation present a fundamental threat to human health. The climate crisis disproportionately affects the poorest and most marginalized, perpetuated by socio-economic gaps, unequal power relations, poor governance and increased risks [5]. This threat is either direct, e.g. via heat stress, flooding, or hurricanes, which present mental and/or physical health issues, or indirect, via the disruption of food systems, increases in zoonoses and food, water and vector borne diseases; and/or because of disruption to the operation of global health systems [6–9]. Certain social groups are particularly vulnerable to these threats, e.g. female-headed households, children, persons with disabilities, indigenous people and ethnic minorities, landless tenants, migrant workers, displaced persons, sexual and gender minorities, older people and other socially marginalized groups [10]. The root causes of their vulnerability lie in a combination of geographical location; financial, socio-economic, cultural and gender status; and access to resources, services, decision-making power and justice. Without effective adaptation and mitigation strategies, such groups are likely to be excluded from digital health systems and benefits, exacerbating preexisting health disparities [11–13]. Digital solutions have been promoted to assist health systems as they mitigate and adapt to climate change and environmental degradation, and in many instances may redress the inequity balance, e.g. by using digital solutions to increase healthcare access to those groups who are marginalized or vulnerable. Telemedicine and the use of drones both represent examples of this [14, 15]. Additionally, digital health tools can provide equitable solutions in humanitarian operations in low-resource and conflict-affected settings. For example, the Pharmacy Stock Management (PSM) system developed by the International Committee of the Red Cross (ICRC) [16], enables health staff to update stock levels using tablets, with automated calculations and order generation to standardize processes and improve visibility across regional and national supply levels. Preliminary findings indicate that the tool has contributed to a reduction of 557kgCO2e/year in carbon emissions through decreased travel, reduced reliance on paper-based systems, minimized stock wastage and streamlined distribution of office and medical supplies. However, it also introduced new sources of carbon impacts, such as 53 kgCO2e/year from data centre usage and by introducing digital tablets. This highlights the potential for digital health tools to enhance service delivery and environmental goals, while at the same time, potentially having their own environmental impacts.

We argue that to fully understand the opportunities and challenges associated with digital health solutions to climate and environment-related issues, the multiple mechanisms that create inequity, including climate and environment, must be understood. We offer four examples to highlight the various inequities that can occur if these issues are not considered throughout the full lifecycle of any digital health technology, from design and development considerations through to monitoring and evaluation. We then provide a series of actionable recommendations for practitioners and policymakers to consider and implement.

## EXAMPLE 1: INEQUALITIES AND E-WASTE

There is an established body of evidence that digital health itself, can add to climate change and environmental degradation, e.g. through rare mineral mining/materials for hardware production, energy consumption during digital hardware usage and digital-related e-waste management and disposal challenges [17, 18]. Taking e-waste as an example, limited regulation exists around disposal, recycling and resource recovery for this type of waste ([19–21], resulting in approximately only one-fifth of e-waste being formally collected and recycled globally. A lack of clarity exists around what happens to the remainder, though it is likely shipped for use in low and middle income (LMICs) countries, abandoned in landfills, or traded through illegal markets [22]. Resource recovery from e-waste landfills in LMICs provides an income source and business opportunities, but unregulated recycling techniques (e.g. open burning, incineration, acid stripping of metals and acid baths) produce hazardous by-products that are present at raised levels in those residing near the informal e-waste sites [19, 23–25].

Efforts to address e-waste place climate justice and health at the heart of digital health solutions. Hameed et al. [26] developed a digital solution to help underserved populations in Sri Lanka’s coastal communities using recycled wearable technologies. With rising temperatures, small-scale fishermen are facing dangerous levels of heat while at sea. This is compounded by limited access to health services and poor social safety nets. Furthermore, fish are becoming increasingly scarce in familiar fishing spots because the fish are migrating to deeper and cooler waters, meaning that the fishermen suffer from many unproductive days in the sea, which in turn, is affecting their mental health. Through community engagement and solidarity-based initiatives, a digital solution to help fishermen was developed. They recycled wearable technologies to offer long-distance emergency communication systems, which reduces the volume of e-waste and extends the life of the product. This includes GPS navigation and real-time weather tracking that allows fishermen to navigate to safer waters, enables health parameter monitoring so that fishermen can monitor their health, and facilitates access to aid in emergency situations through telemedicine.

## EXAMPLE 2: WARNING SYSTEMS & ENVIRONMENTAL APPS

**Figure 1:**
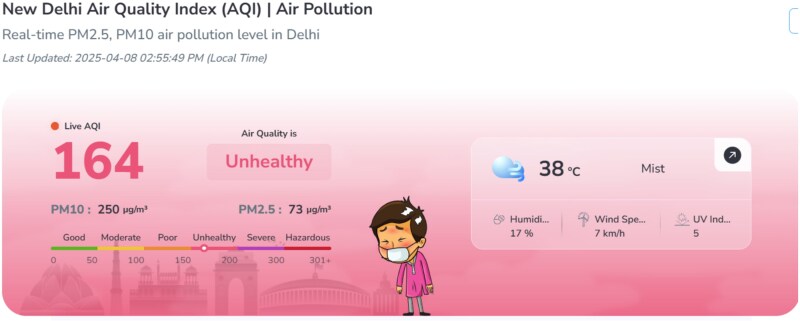
Image from New Delhi air quality index.

Many digital health solutions have been developed to help mitigate and adapt to climate events, such as early warning systems. For example, many smartphone apps are used to inform individuals and communities about environmental conditions, and when necessary, warn of climatic threats. Examples include apps which monitor temperature, humidity, pollution and air quality (e.g. the New Delhi Air Quality Index [27] as seen in Fig. 1, also the Beijing Air Pollution Real-time Air Quality Index [28] and the IQAir AirVisual air quality app [29]. There are app-based early warning systems for climate shocks such as hurricanes and tidal waves, e.g. in Sri Lanka (Sri Lanka Alerts and Sayuru system see [30]).

However, these solutions can further marginalize disadvantaged groups such as those affected by poverty, poor adoption of digital healthcare solutions, lack of access to digital systems and poor digital literacy [31]. This is because these services primarily benefit users of mobile phones; those who do not own or use a mobile phone will not receive the warning apart from indirectly from people who do, or through other channels (e.g. radio, television, sirens) [32]. Several factors are required for people to benefit from these apps. These include ownership of the electronic device, having sufficient data connectivity (incurring financial costs), education and digital literacy and the ability to use and understand the app. The importance of data availability in prediction systems can also put people who generate less data (i.e. people who live in low connectivity areas or people who do not have access to healthcare facilities) in a blind spot, as they contribute less to the datasets that predictions are based on. This creates a representation bias where the input data is not representative for the relevant population, leading to systematic errors in model predictions [33]. In short, these services primarily benefit people who are already digitally empowered.

Approximately 2.9 billion people are still offline and/or do not have access to the internet [32]. Thus, existing inequalities such as the digital divide, necessitate consideration of those without digital connectivity or else there is a risk that these app-based services will compound exclusion as more and more services are only accessible online [32]. While increasing numbers of people are becoming digitally connected, meaning that this problem will reduce over time, many people are likely to remain disconnected for many years still. Ways of addressing this gap in digital access are more social than technical. This can be achieved by ensuring that any climate alert is also broadcast on mainstream media used in the area, especially radio which often has the largest reach; communicating with organisations in the community such as faith-based organisations, political groupings and schools, so that they can notify their members; and encouraging people with connectivity who receive the warnings on their phone to notify others in their community who do not have such access. The use of flashing lights, and loudspeakers on vehicles can also ensure that disconnected groups are reached. Working with local communities for a localized approach is essential, e.g. in Papua New Guinea, Indigenous knowledge as a form of early warning system included the use of a garamut (a large slit drum made from a log) or a conch shell trumpet, which were linked with mobile phone and radio communications from the provincial disaster office [34].

## EXAMPLE 3: VULNERABILITY OF DIGITAL HEALTH SYSTEMS TO CLIMATE EVENTS

Climate change resilience refers to a system’s ability to anticipate, respond to, cope with, recover from and adapt to climate-related shocks and stresses [35]. Health systems, including digital health systems, are heavily dependent on critical, digital and technical infrastructure such as transceiver stations, antennas, data centres, hardware and software, cloud computing, over and underground cables, virtual and physical security measures, as well as electricity and water as critical infrastructure. As the climate changes and we see more weather extremes (e.g. extreme heat or flooding), climate change resilience of the digital health sector is increasingly important. Infrastructure is becoming ever more vulnerable to climate-related effects [36], impacting health care service delivery alongside rescue and recovery efforts and negatively impacting outcomes [37]. This creates risks for digital health systems. The ability to maintain safe and effective care is hindered by extreme weather events which contributes additional damage to unstable energy supplies. An unreliable or disrupted electricity supply is a major barrier to digital connectivity, impacting universal health coverage, resulting in poor health outcomes and inequity [37]. The cost of addressing the direct and indirect impacts of extreme weather is significant [38] and creates additional challenges for health care systems; many facilities in the global south operate under significant financial constraints and already face energy difficulties and frequent blackouts, which create and amplify the existing health inequities. Gogi et al., [37] revealed how flooding in 2024 damaged physical and technical infrastructures in Kenya, cutting off health facilities and submerging them under water as well as interrupting the electricity supply, creating increased demands on the health care system and impacting the public health emergency response. Similarly, Hurricane Elsa in Barbados resulted in power outages due to damage to power lines from fallen trees, resulting in outages of grid-supplied electricity to hospitals. In some cases, patients may be able to move to other facilities, however, damage to digital connectivity can result in a loss of continuity of care due to the lack of integration of electronic health records systems, meaning patient information is not accessible in different geographical areas [17].

**Figure 2:**
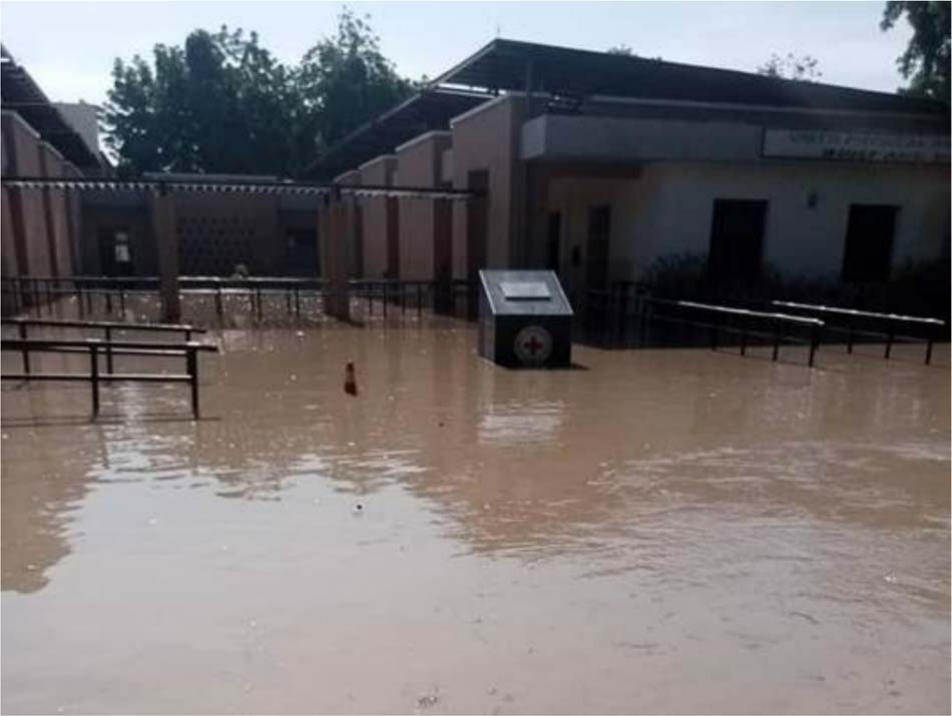
Picture of ICRC supported Maiduguri hospital flooded in Sep 2024. (photographer: Emmanuel Kwaya Daniel, Sep 2024).

**Figure 3:**
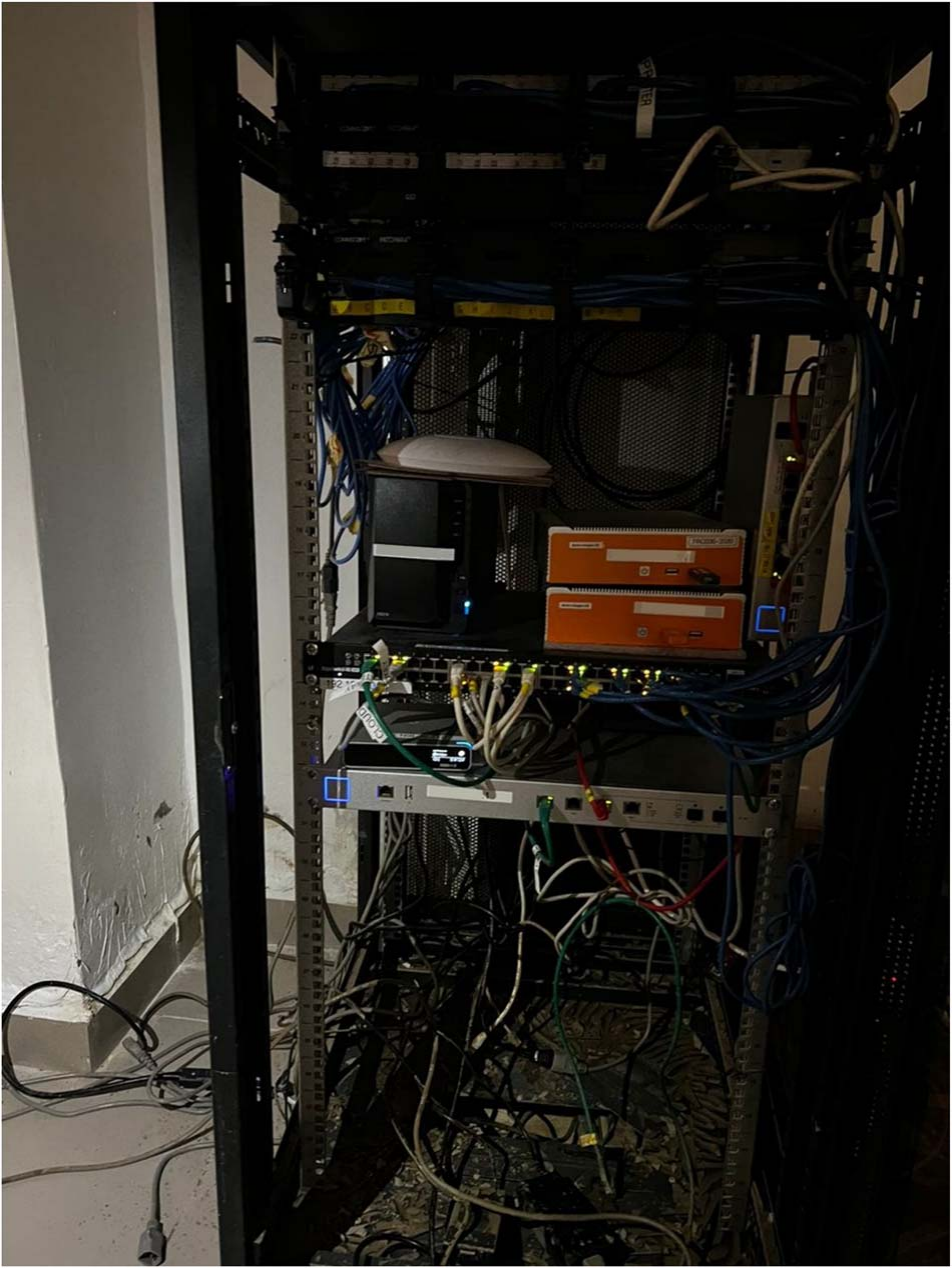
Picture of DCMS boxes (orange) in Maiduguri hospital connected to the rest of the local IT system including visible mud after flood clean up. The water and mud did not rise to the level of the orange boxes but submerged the systems below including one power adapter which led to faults in one of the boxes. (photographer: Emmanuel Kwaya Daniel, Sep 2024).

**Figure 4:**
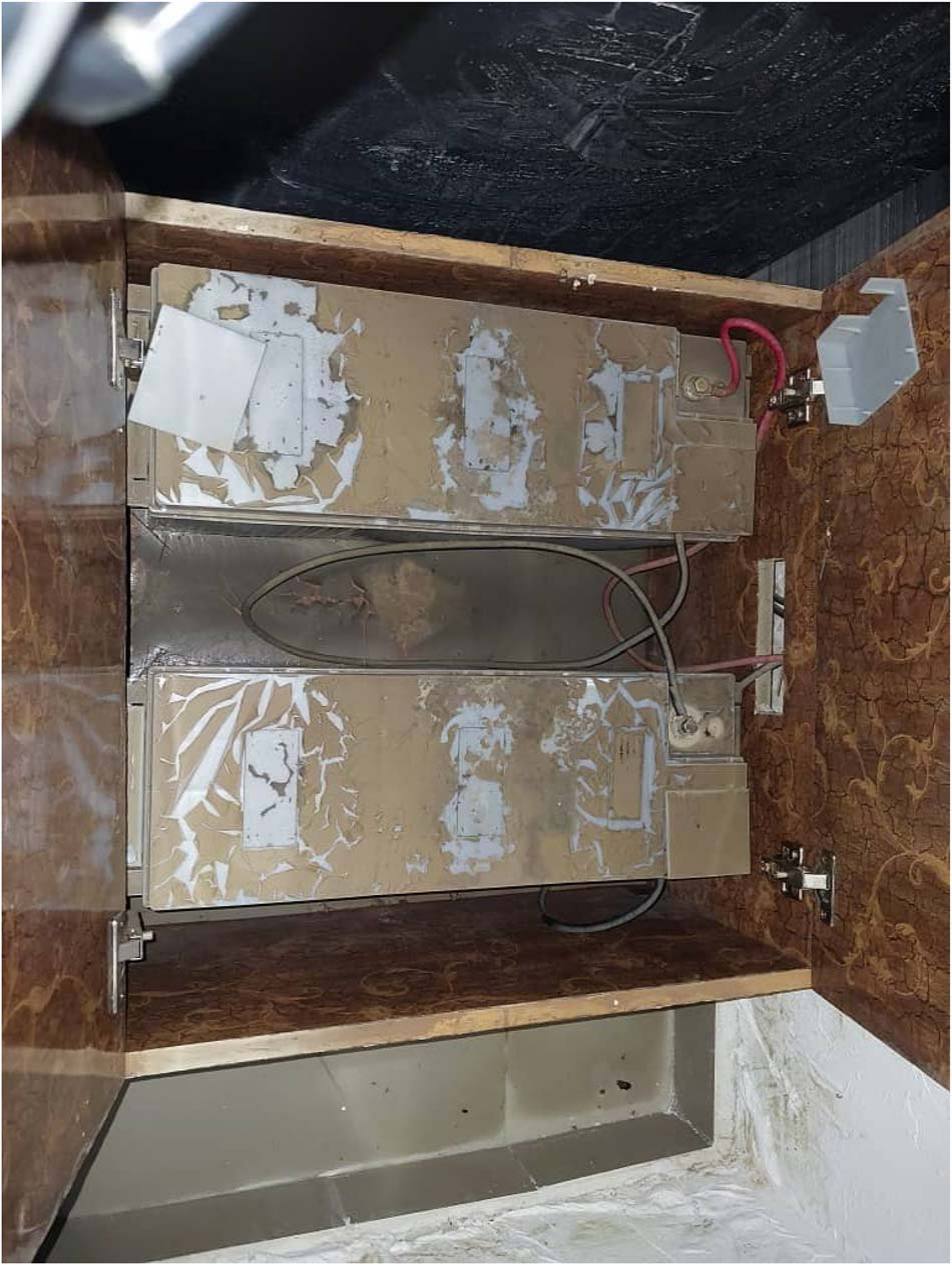
Picture of inverter battery in Maiduguri hospital used to ensure continuous power supply during electricity outages. This is covered in mud after being submerged for several days. The inverter batter remained operational after clean up. (photographer: Emmanuel Kwaya Daniel, Sep 2024).

There are, however, increased efforts to improve the resilience of digital infrastructures and ensure digital health systems remain operational. The WHO [39] cites the need for backup communication systems (such as satellite phones, pagers, two-way radios, alongside emergency generators). The telecom industry is working to maintain climate resilient connectivity, including battery storage solutions which store energy during periods of surplus to supply energy during outages; the use of underground electricity cables which reduces vulnerability to high winds, and replacing wooden or concrete poles with galvanized steel poles to reduce structural damage [37]. Furthermore, the ICRC’s Digital Center Management System (DCMS), which operates in humanitarian settings, offers a relevant example of climate-resilient digital infrastructure for health service delivery in fragile contexts. DCMS is an end-to-end digital platform deployed to support the management of physical rehabilitation centres, hosted on industrial-grade hardware engineered to operate under extreme environmental conditions, including high temperatures, dust and humidity. The selection of this hardware reflects the overlap between environmental and conflict-related risks; regardless of whether damage results from a climate event or conflict-related destruction, the infrastructural resilience requirements remain comparable. In Maiduguri, Borno State, Nigeria, DCMS had been implemented at a hospital before the collapse of the Alau Dam on the 10^th^ September 2024, which led to extensive flooding and displaced over 70% of the city’s population, see Fig. 2 for the scale of flooding the hospital faced. During the rapid evacuation, the hardware remained onsite and was submerged in mud for several days. Despite these adverse conditions, parts of the system remained fully functional and resumed operations immediately following cleanup, with no data loss and minimal technical failure, see Fig. 3 and Fig. 4. Ultimately, placing key systems on elevated shelving as a precautionary measure protected them from flood damage and others remained functional due to their rugged designs. On the other hand, simple items like power adapters could disrupt more advanced parts of the system. This case highlights the importance of designing durable, context-appropriate digital systems capable of withstanding acute climate-related shocks while ensuring continuity of essential health services. It is also a reminder that the system is as strong as its weakest link and a whole system approach needs to be considered to ensure maximum resilience. Thus there is a need to combine low-cost strategies like equipment location and placement with higher-cost design choices appropriate for the context.

## EXAMPLE 4: ENVIRONMENTAL IMPACTS OF ARTIFICIAL INTELLIGENCE

The environmental impacts of artificial intelligence (AI) have been gathering increased attention, including the need for critical minerals for hardware and that AI models consume vast amounts of electricity [40]. Additionally, during construction and use, data centres that house data for AI models use large amounts of water to cool electrical components. Frank and News [41] noted that it is estimated that AI-related infrastructures may consume more than six times more water than Denmark, with its population of 6 million people. As AI systems become more complex and require more computing power, it has been argued that the amount of water required to cool data centres will also increase, which could result in water scarcity, increased energy use and increased greenhouse gas emissions [42]. The negative impact of AI often disproportionately affects regions particularly vulnerable to climate and other related harms [43]. Data centres are often located in areas close to low-income communities, meaning it is these communities who may have water scarcity and or biodiversity loss [44]. Fossil fuel-reliant data centres contribute to global warming, which most affect communities in low-income countries and small island developing states because of their lack of resources to adapt, even though they contribute/contributed least to global emissions.

Efforts to develop green or sustainable AI are being considered through optimisation of algorithms for efficiency to reduce computational resource requirements [45]. Other solutions include the use of solar farms, the procurement of energy credits and commitments to replenish more water than data centres use, and data centre optimisation, whereby algorithms and frameworks dynamically manage server loads, adjust cooling systems and optimize resource allocation to reduce energy consumption [41]. Guidelines should be developed for healthcare staff, researchers, AI developers and policymakers to harness AI’s potential while mitigating its environmental harms as much as possible [46, 47]. These should be integrated with broader sustainability initiatives within healthcare organisations and systems by aligning AI sustainability with institutional sustainability [47]. At the same time, it is important to also consider the unintended rebound effects associated with AI. Rebound effects occur when energy savings associated with increases in (AI) efficiency are less than expected because of behaviour change associated with efficiency changes (i.e. ‘the algorithm is more efficient, so I can run more algorithms’). In extreme cases, rebound effects backfire when consumption increases rather than decreases. Backfire is evident in the digital sector, which has witnessed increased energy consumption over the decades despite increases in efficiency [48, 49].

## RECOMMENDATIONS

**Figure 5:**
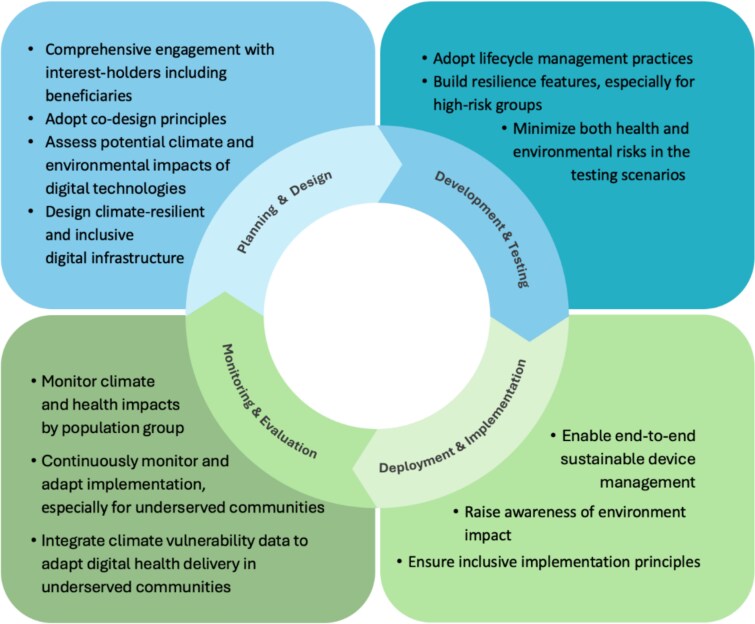
Diagrammatic summary for integrating equity and climate resilience across the digital health lifecycle.

To ensure equity in the digital health system, we recommend the following. These have been summarized visually in Fig. 5 to assist the digital health community in implementing the recommendations into policy and practice:

- When designing and delivering digital solutions, equity analyses should be considered as an integrated part throughout the digital health lifecycle. This should involve comprehensive stakeholder engagement, particularly with marginalized communities, to understand specific vulnerabilities and barriers to digital access and to minimize those barriers.
- Prioritising sustainable investment in the development of climate and environment resilient digital health infrastructure, including energy backup systems, secure data storage, robust communication networks and offline capabilities. Designing digital health systems with these resilience features from the outset is essential to ensure continuity of care during extreme climate events.
- Including a well-organized plan for improving digital literacy, subsidizing connectivity costs and creating offline solutions for low-resource settings to ensure inclusion of disadvantaged populations in all digital health strategies.
- Including specific guidelines for managing e-waste, promoting recycling and resource recovery in national and international digital health policies, especially in low- and middle-income countries.
- Ensuring manufacturers and healthcare providers adopt lifecycle management practices to minimize environmental impacts. It is also necessary to ensure continuous education for healthcare professionals and policymakers regarding environmental impacts and sustainability practices to further reinforce these efforts.
- Having policy implementation plans which monitor and evaluate the impact of the digital health technologies for communities should be planned and enacted. Adoption of policy frameworks which allow these impacts to be recognized and acted upon by the government/interest-holders of the affected communities, to mitigate the impact of the digital health solutions.

## References

[1] Olu O, Muneene D, Bataringaya JE et al. How can digital health technologies contribute to sustainable attainment of universal health coverage in Africa? A perspective. *Front Public Health* 2019;7:341. 10.3389/fpubh.2019.00341 31803706 PMC6873775

[2] WHO . Global strategy on digital health 2020–2025. Geneva: World Health Organization, 2021.

[3] WHO *Health Equity.* 2025 Available online; https://www.who.int/health-topics/health-equity#tab=tab_1. (4 June 2025, date last accessed).

[4] Braveman P, Gruskin S. Defining equity in health. *J Epidemiol Community Health* 2003;57:254–8. 10.1136/jech.57.4.254 12646539 PMC1732430

[5] Bhargawa R, Bhargava M, *The climate crisis disproportionately hits the poor. How can we protect them*? 2023 Available online: https://www.weforum.org/stories/2023/01/climate-crisis-poor-davos2023/. (30 June 2025, date last accessed).

[6] Ebi KL, Hess JJ. Health risks due to climate change: inequity in causes and consequences: study examines health risks due to climate change. *Health Aff* 2020;39:2056–62. 10.1377/hlthaff.2020.01125 33284705

[7] Ebi KL, Vanos J, Baldwin JW et al. Extreme weather and climate change: population health and health system implications. *Annu Rev Public Health* 2021;42:293–315. 10.1146/annurev-publhealth-012420-105026 33406378 PMC9013542

[8] WHO ‘*Climate Change'*. 2023a Available online: https://www.who.int/news-room/fact-sheets/detail/climate-change-and-health#:∼:text=Climate%20change%20is%20impacting%20health,diseases%2C%20and%20mental%20health%20issues. (25 February 2025, date last accessed).

[9] WHO *COP29 Special report and Climate Change and Health.* Health is the argument for climate action. 2024 Available online; https://cdn.who.int/media/docs/default-source/environment-climate-change-and-health/58595-who-cop29-special-report_layout_9web.pdf?sfvrsn=dd2b816_8. (25 February 2025, date last accessed).

[10] Zahnow R, Yousefnia AR, Hassankhani M et al. Climate change inequalities: a systematic review of disparities in access to mitigation and adaptation measures. *Environ Sci Pol* 2025;165:104021. 10.1016/j.envsci.2025.104021

[11] Coetzer JA, Loukili I, Goedhart NS et al. The potential and paradoxes of eHealth research for digitally marginalised groups: a qualitative meta-review. *Soc Sci Med* 2024;350:116895. 10.1016/j.socscimed.2024.116895 38710135

[12] Ngcamu BS . Climate change effects on vulnerable populations in the global south: a systematic review. *Nat Hazards* 2023;118:977–91. 10.1007/s11069-023-06070-2

[13] Putsoane T, Bhanye JI, Matamanda A. Extreme weather events and health inequalities: exploring vulnerability and resilience in marginalized communities. *Dev Environ Sci* 2024;15:225–48. 10.1016/B978-0-443-21948-1.00011-X

[14] O'Sullivan B, Leaman M. Medical delivery drones as a tool to improve health equity in sub-Saharan Africa. *Global Health: Ann Rev* 2022;1:70–3.

[15] Whaibeh E . The good, the bad, and the disruptive: how telehealth can reduce health inequity and mitigate the digital divide in the MENA region. *Medicine, Conflict and Survival* 2022;38:339–45. 10.1080/13623699.2022.2139871 36317382

[16] Harris F, Beau R, Elkin J. Exploring the carbon impact of the use of a digital pharmacy stock management tool in Nigeria and Somalia [Conference poster] Global Digital Health forum, Nairobi, Kenya, 2024

[17] Lokmic-Tomkins Z, Davies S, Block LJ et al. Assessing the carbon footprint of digital health interventions: a scoping review. *J Am Med Inform Assoc* 2022;29:2128–39. 10.1093/jamia/ocac196 36314391 PMC9667173

[18] Ogunseitan OA . Side effects of the electronic health care revolution: toxic E-waste. *World Neurosurg* 2022;167:2–3. 10.1016/j.wneu.2022.07.124 35948224

[19] Gabrys J . Digital rubbish: A natural history of electronics. Ann Arbor, MI: University of Michigan Press, 2013.

[20] Lepawsky J . Reassembling Rubbish: Worlding Electronic. Cambridge, MA: The MIT Press, 2018.

[21] Mmereki D, Baldwin A, Li B. A comparative analysis of solid waste Management in Developed, developing and lesser developed countries. *Environ Technol Rev* 2016;5:120–41. 10.1080/21622515.2016.1259357

[22] Forti V, Baldé CP, Kuehr R et al. *The global e-waste monitor 2020: Quantities, flows and the circular economy potential*. United Nations University (UNU), International Telecommunication Union (ITU), and International Solid Waste Association (ISWA). 2020 Available online: https://www.itu.int/en/ITU-D/Environment/Documents/Toolbox/GEM_2020_def.pdf. (10 November 2025, date last accessed).

[23] Dai Q, Xu X, Eskenazi B et al. Severe dioxin-like compound (DLC) contamination in e-waste recycling areas: an under-recognized threat to local health. *Environ Int* 2020;139:105731. 10.1016/j.envint.2020.105731 32315892

[24] Ngo HTT, Watchalayann P, Nguyen DB et al. Environmental health risk assessment of heavy metal exposure among children living in an informal E-waste Processing Village in Vietnam. *Sci Total Environ* 2021;763:142982. 10.1016/j.scitotenv.2020.142982 33129545

[25] Singh N, Ogunseitan OA, Tang Y. Systematic review of pregnancy and neonatal health outcomes associated with exposure to e-waste disposal. *Crit Rev Environ Sci Technol* 2020;51:2424–48. 10.1080/10643389.2020.1788913

[26] Hameed I , Empowering Coastal Communities through Recycled Wearable Technologies for Climate Justice and Health- A Highlight at the GDHF Lightening Talk. 2024 Available online: https://inorder.in/blog-fields/empowering-coastal-communities-through-recycled-wearable-technologies-for-climate-justice-and-health-a-highlight-at-the-gdhf-lightening-talk/. (7 July 2025, date last accessed).

[27] AQI Air Quality Index. n.d. Available online: https://www.aqi.in/dashboard. (7 July 2025, date last accessed).

[28] Beijing Environmental Protection Monitoring Center Beijing Air Pollution: Real-time Air Quality Index (AQI). n.d. Available online: https://aqicn.org/city/beijing/. (7 July 2025, date last accessed).

[29] IQ AIR Air Quality App. n.d. Available online https://www.iqair.com/gb/air-quality-monitors/air-quality-app. (7 July 2025, date last accessed).

[30] Radjy S. Early warning and location technology saves lives in Sri Lanka. 2024 Available online: https://www.preventionweb.net/news/early-warning-and-location-technology-saves-lives-sri-lanka. (7 July 2025, date last accessed).

[31] Perera D, Agnihotri J, Seidou O et al. Identifying societal challenges in flood early warning systems. *Int J Disaster Risk Reduct* 2020;51:101794. 10.1016/j.ijdrr.2020.101794

[32] ITU Digital transformation and early warning systems for saving lives. 2023 Available online https://www.itu.int/hub/publication/d-gen-digital-transfor-01-2023/. (30 June 2025, date last accessed).

[33] Van Giffen B, Herhausen D, Fahse T. Overcoming the pitfalls and perils of algorithms: a classification of machine learning biases and mitigation methods. *J Bus Res* 2022;144:93–106. 10.1016/j.jbusres.2022.01.076

[34] United Nations Office for Disaster Risk Reduction, World Meteorological Organization Centre of Excellence for Climate and Disaster Resilience . Early Warning Systems and Early Action in Fragile, Conflict-affected and Violent Contexts: Addressing Growing Climate and Disaster Risks. Geneva, 2024.

[35] WHO , *Operational Framework for Building Climate Resilient and Low Carbon Health Systems.* 2023b Available online: https://iris.who.int/bitstream/handle/10665/373837/9789240081888-eng.pdf?sequence=1. (24 March 2025, date last accessed). 10.1136/bmj.p2856 38052463

[36] Palmer K , *Unpacking the vicious cycle of climate change and digital security*, 2025 Available online: https://bindinghook.com/articles-binding-edge/unpacking-the-vicious-cycle-of-climate-change-and-digital-security/. (24 March 2025, date last accessed).

[37] Gogi E, Luthra R, Vaish S et al. *Climate Resilience and Powering Healthcare in the Global South.* 2025 Available online: https://www.seforall.org/system/files/2025-02/SEforALL-phc-climate-resilience-study.pdf. (4 June 2025, date last accessed).

[38] Newman R, Noy I. The global costs of extreme weather that are attributable to climate change. *Nat Commun* 2023;14:6103. 10.1038/s41467-023-41888-1 37775690 PMC10541421

[39] WHO . WHO guidance for climate-resilient and environmentally sustainable health care facilities. Geneva: World Health Organization, 2020. 10.3390/ijerph17238849 PMC7731282 33260752

[40] UNEP , *AI has an environmental problem Here’s what the world can do about that* 2024 Available online: https://www.unep.org/news-and-stories/story/ai-has-environmental-problem-heres-what-world-can-do-about. (4 June 2025, date last accessed).

[41] Frank T, News EE. A*I’s Climate Impacts May Hit Marginalized People Hardest.* 2024) Available online; https://www.scientificamerican.com/article/ais-climate-impacts-may-hit-marginalized-people-hardest/#:∼:text=AI's%20climate%20impacts%20are%20heaviest,and%20access%20to%20potable%20water.%E2%80%9D. (23 March 2025, date last accessed).

[42] George AS, George AH, Martin AG. The environmental impact of AI: a case study of water consumption by chat gpt. *Partners Univ Int Innov J* 2023;1:97–104. 10.5281/zenodo.7855594

[43] Ren S, Wierman S. *The Uneven Distribution on AI’s Enviornmental Impacts*, 2024 Available online: https://hbr.org/2024/07/the-uneven-distribution-of-ais-environmental-impacts. (23 March 2025, date last accessed).

[44] Barrett L, Gambarini C. Revealed: *Big tech’s new datacentres will take water from the world’s driest areas.* 2025 Available online: https://www.theguardian.com/environment/2025/apr/09/big-tech-datacentres-water. (4 June 2025, date last accessed).

[45] Bolón-Canedo V, Morán-Fernández L, Cancela B et al. A review of green artificial intelligence: towards a more sustainable future. *Neurocomputing* 2024;599:128096. 10.1016/j.neucom.2024.128096

[46] Lannelongue L, Aronson HEG, Bateman A et al. GREENER principles for environmentally sustainable computational science. *Nat Comput Sci* 2023;3:514–21. 10.1038/s43588-023-00461-y 38177425

[47] Ueda D, Walston SL, Fujita S et al. *Climate change and artificial intelligence in healthcare*: review and recommendations towards a sustainable future. *Diagn Interv Imaging* 2024;105:453–9. 10.1016/j.diii.2024.06.002 38918123

[48] Hilty LM, Köhler A, Von Schéele F et al. Rebound effects of progress in information technology. *Poiesis Prax* 2006;4:19–38. 10.1007/s10202-005-0011-2

[49] Widdicks K, Lucivero F, Samuel G et al. Systems thinking and efficiency under emissions constraints: addressing rebound effects in digital innovation and policy. *Patterns* 2023;4:100679. 10.1016/j.patter.2023.100679 36873905 PMC9982294

